# Pangenomes of the human oral microbiome

**DOI:** 10.1128/mra.00261-26

**Published:** 2026-05-29

**Authors:** Julian Torres-Morales, Jonathan J. Giacomini, Tsute Chen, Andrew Voorhis, Kathryn M. Kauffman, Floyd E. Dewhirst, Gary G. Borisy, Jessica L. Mark Welch

**Affiliations:** 1 ADA Forsyth Institute, Somerville, Massachusetts, USA; 2 Marine Biological Laboratory 42700, Woods Hole, Massachusetts, USA; 3 Department of Oral Biology, School of Dental Medicine, The University at Buffalo 551149 , Buffalo, New York, USA; 4 Harvard School of Dental Medicine 124048 , Boston, Massachusetts, USA; DOE Joint Genome Institute, Berkeley, California, USA

**Keywords:** pangenomes, human microbiome, oral microbiome, large-scale genomics, comparative genomics

## Abstract

We announce the release of 579 pangenomes derived from 8,115 genomes curated by the Human Oral Microbiome Database, capturing shared and variable gene content across oral microbial taxa. This openly accessible resource supports both online and offline exploration, enabling systematic studies of microbial function, evolution, and community structure.

## ANNOUNCEMENT

**Fig 1:**
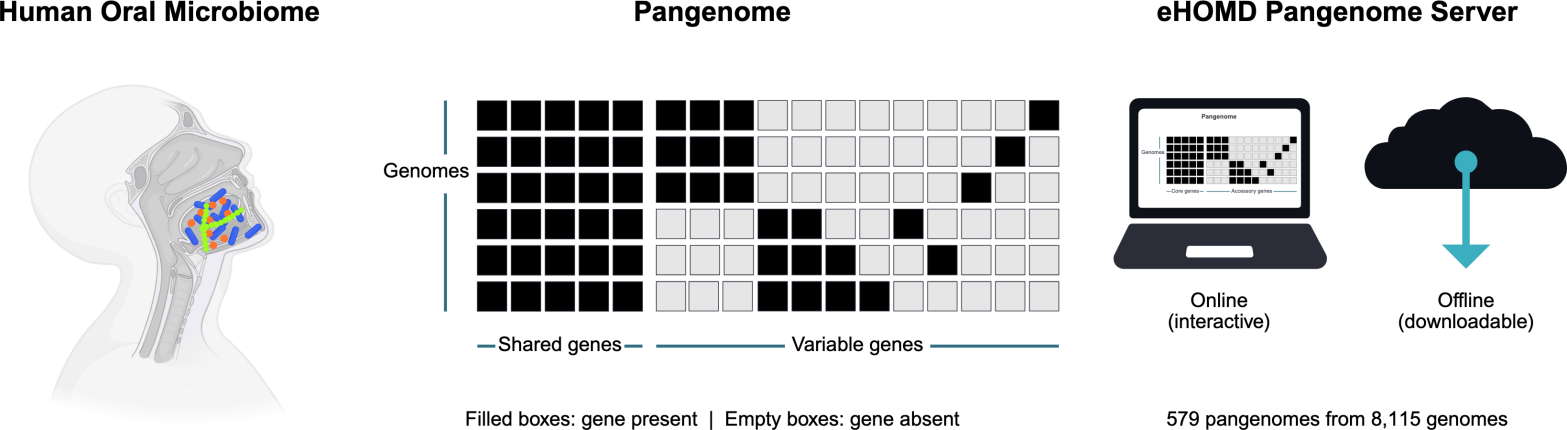
Conceptual overview of human oral microbiome pangenomes. Strain-level diversity within the oral microbiome is captured in 579 taxon-resolved pangenomes spanning 8,115 genomes, highlighting shared and variable gene clusters that embed an evolutionary and ecological context. Pangenomes are explorable via an eHOMD Anvi’o portal and available for offline reuse, providing a resource to study functional and ecological variation across oral taxa. Created in BioRender. Torres-Morales, J. (2026) https://BioRender.com/47493js.

The human oral microbiome comprises hundreds of microbial taxa that collectively shape oral and systemic health (1–4). Here, we announce a comprehensive pangenomic resource capturing strain-level genomic diversity across the human oral microbiome. Genomic variation at the strain level underpins functional capacity and microbe-microbe and host-microbe interactions (5–8) (Fig. 1). Systematic investigation of this fine-scale diversity has remained limited (9, 10), largely because standardized and accessible pangenomic resources have been lacking.

To address this gap, we constructed and released pangenomes for taxa represented in the expanded Human Oral Microbiome Database (eHOMD) (11, 12), available at https://www.homd.org/genome/anvio_pangenomes. We define a pangenome as the complete repertoire of protein-coding genes encoded across genomes assigned to a human microbial taxon (HMT) curated in eHOMD based on 16S rRNA and whole-genome sequences (11). Within this framework, genes predicted in each genome are grouped into homologous gene clusters representing evolutionarily related sequences. These clusters may be widely shared, variably distributed, or strain specific. By integrating quality-filtered genomes curated by eHOMD from cultured isolates, single-cell and metagenome-assembled genomes, and long-read assemblies, this resource captures functional and evolutionary variation across eHOMD taxa, including oral residents and other taxa potentially detected in oral samples.

We generated 579 pangenomes, including 567 HMT-level pangenomes representing taxa with two or more genomes, and 12 multi-clade species-level pangenomes integrating genomes from multiple HMTs. The 567 HMT-level pangenomes comprise 8,110 genomes, while five additional genomes from single-genome HMTs were included exclusively in the species-level pangenomes, totaling 8,115 genomes. Together, these represent 99.2% of genomes in eHOMD (v4.1 φ, Genomes v11.2). Pangenomes are distributed through a dedicated eHOMD-hosted server, enabling software-free exploration alongside reproducible offline use.

Genomes were processed using two custom Snakemake (13) workflows leveraging Anvi’o (v8) (14–16), tools selected for reproducibility and gene-centric visual interpretation. The first workflow generates genome-level “contig” databases and performs annotations, including prediction of rRNAs, tRNAs, universal single-copy genes, COGs (17), KEGG orthologs (18), Pfam domains (19), and carbohydrate-active enzymes (20). The second workflow constructs pangenomes, computes pairwise average nucleotide identity, estimates metabolic potential, and reconstructs phylogenies from single-copy core gene clusters, linking gene content variation with evolutionary relationships.

Homologous gene clusters were delineated using pairwise amino acid similarity with a minbit threshold of 0.5 (BLASTP bit score/self-alignment score ≥0.5) and the Markov clustering algorithm inflation = 10 (21). Gene clusters and genomes are hierarchically organized by gene-cluster presence-absence patterns, enabling exploration of shared and variable genomic features across strains.

All pangenomes are provided as pre-computed Anvi’o databases via an eHOMD-hosted server. Users can interactively explore gene-cluster distributions and predicted functions without local software installation, while offline access supports advanced, customizable analyses.

By uniting biological depth with technical accessibility and visual interpretability, this resource provides a foundation for studying microbial diversity across the human oral microbiome. The framework is extensible to other ecosystems and will expand as additional high-quality genomes—including long-read assemblies—become available (22). By democratizing access to high-resolution pangenomes, this resource empowers discovery, comparative genomics, and education at the ecosystem scale.

This comprehensive pangenomic resource transforms strain-level diversity of the human oral microbiome into an accessible, standardized, and reusable framework, enabling biological discovery at a scale previously inaccessible to the field.

## Data Availability

Access to the Pangenome eHOMD server online and downloadable summary files (tarball and SHA-256 checksum) is available at the same portal (https://www.homd.org/genome/anvio_pangenomes). All workflows are fully documented and publicly available on GitHub (https://github.com/juliantom/Pangenomes_of_the_Human_Oral_Microbiome_Database).
